# The Humanity of Animal Experimentation

**Published:** 1910-03

**Authors:** 


The Humanity of Animal Experimentation.—Millions of animals, birds, and fishes are tortured and slain every year to provide food, clothing, and mere sport for mankind, and the zoophilists say nothing, but when a few hundred or a thousand animals are sacrificed for the sake of knowledge that will save the lives of countless children and avert destructive epidemics, a cry of pain goes up, and the lawmakers are prayed to arrest the progress of medical science. They will not do it, of course, they can not do it, for the great mass of humanity is sane, but the periodical agitation against animal experimentation is none the less distressing to the lovers of their kind.—*New York Medical Journal.*

